# Review and analysis of knowledge, tools, and implementation gaps for the control of cryptosporidiosis in livestock

**DOI:** 10.1186/s12917-026-05779-z

**Published:** 2026-08-17

**Authors:** Lucy J. Robertson, Guy Robinson, Simone M. Cacciò, Kurt Hanevik, Monica Santin, Karin Troell, Mark van Roosmalen, Geert Vertenten, Frank Katzer

**Affiliations:** 1 Parasitology, Faculty of Veterinary Medicine, Norwegian University of Life Sciences, Postbox 5003, Ås, 1432 Ås Norway; 2 Cryptosporidium Reference Unit, Public Health Wales, Singleton Hospital, Swansea, SA2 8QA UK; 3 Swansea University Medical School, Singleton Park, Swansea, SA2 8PP UK; 4 Department of Infectious Diseases, Istituto Superiore di Sanità, Rome, Italy; 5 Department of Clinical Science, University of Bergen, Bergen, Norway; 6 Environmental Microbial and Food Safety Laboratory, Agricultural Research Service, Department of Agriculture, Beltsville, MD 20705 USA; 7 Norwegian Veterinary Institute, Ås, Norway; 8 MSD Animal Health, Boxmeer, 5831 AN The Netherlands; 9 Moredun Research Institute, Pentlands Science Park, Edinburgh, EH26 0PZ UK

**Keywords:** *Cryptosporidium parvum*, Calves, Cattle, Colostrum, Diagnostic, Diarrhoea, Farm management, Ruminants, Treatment, Vaccine

## Abstract

Cryptosporidiosis, the disease caused by infection with *Cryptosporidium* spp., is an important threat to both human and veterinary health; some *Cryptosporidium* species, particularly *Cryptosporidium parvum*, being zoonotic. The most usual manifestation of cryptosporidiosis is acute diarrhoeal disease, but respiratory disease due to *Cryptosporidium baileyi* infection can also occur in poultry. In other livestock, particularly young ruminants in which *C. parvum* is the most clinically relevant species, this can have an important impact. Although the mortality rate is generally low, morbidity can be considerable and can result in significant economic losses due to veterinary costs and reduced productivity. Considerable efforts have therefore been directed towards developing tools for combating cryptosporidiosis in livestock. Although diagnostic tools have improved in the past decades, treatments and prevention have not advanced at a commensurate pace. The two mainly available pharmaceutical treatments for livestock are shown to have some effect on reducing diarrhoea and oocyst shedding, but have limited therapeutic windows and restrictions on use. A recently introduced vaccine provides protection to calves by raising antibodies against *C. parvum* in pregnant cows, which are transferred to their calves via colostrum. It is only for use in cattle and not available globally. With both the available pharmaceutical treatments and the vaccine, oocyst shedding is not eliminated, thus environmental contamination remains a source of infection for animals and people. This means that on-farm operations, including biosecurity measures and management of slurry and manure, should be used in combination with pharmaceutical treatment and vaccination. However, the efficacy of such measures needs further investigation.

This review is based on the recently updated DISCONTOOLS analysis, which identifies knowledge gaps and advances the development and implementation of new tools for controlling cryptosporidiosis in livestock. While vaccination and treatment have important roles, appropriate farm management, especially good farm hygiene, as well as handling and treatment of manure, remains a vital component for reducing the risk of cryptosporidiosis.

## Introduction

Cryptosporidiosis is generally characterized as a diarrhoeal disease in a wide range of animals, including mammals and birds, following infection with various *Cryptosporidium* species, some of which are host specific and some of which have a range of different potential hosts. Some species are zoonotic or have zoonotic potential, whereas others have not been detected as infections in people [1]. In general, infection elicits a strong immune response, protecting against disease and future infections [2], which means that cryptosporidiosis is generally seen as a self-limiting disease in neonates, the very young, those with no prior exposure, or those with weakened immune systems. Among livestock, both ruminants (cattle, sheep, goats, deer) and pseudo-ruminants (llamas, alpacas, camels) seem to be particularly affected, but other livestock species, including pigs, rabbits, and poultry can also be infected and develop clinical disease [3].

In terms of production losses, both in terms of mortality and morbidity, cattle have been particularly affected by cryptosporidiosis, with one *Cryptosporidium* species, *C. parvum*, predominating in terms of development of clinical disease, and zoonotic transmission [2, 4]. In recognition of its importance as a major disease of livestock, cryptosporidiosis was included in the DISCONTOOLS database, which aims “to identify the gaps in knowledge to speed up the development of new disease control tools (diagnostics, vaccines and pharmaceuticals) and to reduce the burden of animal diseases” in 2018. During 2024, it was decided that due to acquisition of new knowledge, and important new advances in control, the information on the database should be re-evaluated and updated. A group of experts (all the authors of this article) was convened to undertake this task, using a standardised set of questions, to consolidate current knowledge and (research) gaps on cryptosporidiosis and its control tools. The full assessment details of this output are freely available at https://www.discontools.eu/database/52-cryptosporidiosis.html [5]. In the DISCONTOOLS database, cryptosporidiosis (along with many other diseases of livestock) is presented as an overall “Disease and product analysis” with five sections (Control tools, Disease details, Global challenges, Main critical gaps, Conclusion. Sources of information). In addition, it is possible to look at a Prioritization Model, a Gap Analysis, and Scoring criteria (which can be for just cryptosporidiosis or several diseases simultaneously). The intention is that by identifying gaps in both knowledge and available control tools, research can be prioritized to accelerate the development of new diagnostics, vaccines, and pharmaceuticals, thereby reducing the burden of animal diseases.

In the current article, we provide an overview of the available information on livestock cryptosporidiosis, its aetiology, pathogenesis, epidemiology, impact, and control. In particular, we identify the main gaps in knowledge, tools, and other requirements necessary to improve the control of cryptosporidiosis. While infection with different *Cryptosporidium* species, potentially resulting in clinical cryptosporidiosis, occurs in a range of animals, the focus of this article is on *C. parvum.* This *Cryptosporidium* species causes the greatest disease burden among livestock and is of significant zoonotic importance [2, 4]. Our particular emphasis is on infection in cattle.

## *Cryptosporidium*: the pathogen and its transmission and impact

### Overview of *Cryptosporidium* lifecycle and morphology

The *Cryptosporidium* genus is classified within the phylum Apicomplexa and comprises many species of obligate intracellular parasites that infect a broad range of vertebrate hosts including mammals, reptiles, birds, fish and amphibians [1].

The life cycle starts with the ingestion of *Cryptosporidium* oocysts, which are small (mostly 4–6 μm in diameter), but extremely robust. Oocysts in the host intestine excyst, releasing four sporozoites per oocyst. These are motile, and they contact and actively invade epithelial cells, where they occupy a peculiar intracellular but extra-cytoplasmatic niche. After several rounds of asexual replication that result in destruction of the cells that are hosting *Cryptosporidium*, the parasite enters the sexual phase of the cycle. This culminates in the production of new oocysts that are shed with the host faeces and are immediately infectious to the next host on shedding [6].

### Host range, including zoonotic infection

The *Cryptosporidium* genus comprises over 40 species with considerable differences in host specificity. In humans, *C. hominis* and *C. parvum* are the primary pathogens. The latter has a demonstrated zoonotic potential [7] and is able to infect livestock species, in particular calves, but also lambs and goat kids. In poultry, cryptosporidiosis presents as respiratory disease in chickens and other galliforms, and as intestinal disease in turkeys and quails [8]. Among the species causing avian cryptosporidiosis, *C. meleagridis* is recognized as an important human pathogen.

### Transmission routes

*Cryptosporidium* oocysts are thick-walled and highly resistant to environmental stress and can survive for months in cool, moist conditions but are susceptible to desiccation [9]. Oocysts also retain their infectivity when exposed to standard chlorine concentrations used for water treatment.

Transmission occurs primarily through the faecal-oral route, with infected hosts shedding very large numbers of oocysts, reaching up to millions of oocysts per gramme of faeces [3]. The infective dose is low, with as few as 10–100 oocysts capable of causing disease in susceptible hosts [10, 11].

Seasonal patterns influence transmission dynamics, with calving and lambing periods increasing transmission risk. However, it has also been observed that *Cryptosporidium* prevalences were highest in autumn for all animal categories [12].

### Geographical distribution

*Cryptosporidium* has a global distribution, but species distribution and prevalence vary by region. In high-income countries, human infections with *C. parvum* are at least as common as those caused by *C. hominis*, while in low-income countries, particularly in sub-Saharan Africa, *C. hominis* largely predominates in people [13]. Similarly, although *C. parvum* is the predominant species infecting young ruminants in Europe, North America, and many other regions, this is not the case globally. For example, in sub-Saharan African countries, *C. parvum* is currently relatively seldom found infecting livestock, where less pathogenic *C. xiaoi*,* C. bovis* and *C. ubiquitum* tend to predominate [13] and pre‑weaned calves in China are more commonly infected with *C. bovis* [14].

### Impacts on human health and farm-level/productivity losses

In immunocompetent persons, cryptosporidiosis is characterized by acute diarrhoea and abdominal pain, but symptoms are usually self-limiting. On the other hand, infection is more severe, sometimes fatal, in naïve or immunocompromised hosts.

Our understanding of the impact of cryptosporidiosis on human health has significantly improved in the last two decades. The GEMS study found *Cryptosporidium* to be one of the most important pathogens causing diarrhoea in young children in low-income countries [15] and the annual number of *Cryptosporidium*-attributable diarrhoea episodes in children has been estimated to be 2.9–4.7 million [16]. Several studies have highlighted that *Cryptosporidium* is associated with raised levels of inflammatory markers and growth faltering, particularly stunting, following infection [17–20]. Considering both the acute and long-term effects of cryptosporidiosis, the most recent estimates of health burden due to cryptosporidiosis have been reported by the Institute for Health Metrics and Evaluation to be 60,772 deaths and 4.65 million DALYs per year in 2023, which is higher, for example, than the health burden from non-typhoidal *Salmonella* (27,979 deaths, 1.69 million DALYs) [21].

Likewise, the burden of disease is much higher in very young animals that experience severe cryptosporidiosis, resulting in significant economic losses due to mortality and reduced growth rates caused by disease morbidity. In a Scottish suckler herd, a reduction in growth resulted in a weight difference of 34 kg between clinically affected and unaffected calves when they were 6 months of age, which was estimated to be a loss of £130 per calf [4]. Young ruminants can become infected and begin shedding *Cryptosporidium* oocysts shortly after birth, with the highest rates of infection reported in animals between 1 and 3 weeks of age [22]. The impact of cryptosporidiosis may be aggravated in cases where there are concurrent infections with other enteropathogens [23]. A study conducted in several European countries estimated an average loss of €40 per calf due to cryptosporidiosis [24]. It has been suggested that cryptosporidiosis in the early life of a dairy cow can lead to subsequent reductions in milk yields, but empirical studies are required to confirm this and to estimate the associated production losses [24].

## Cryptosporidiosis: the disease in livestock

Although, infection with *Cryptosporidium* frequently results in cryptosporidiosis, predominantly presenting as a diarrhoeal disease in livestock, there is considerable variation between livestock species, and also in the disease spectrum and organs affected associated with a particular *Cryptosporidium* species within the host livestock type. Different age groups may also be more pre-disposed to infection with different *Cryptosporidium* species.

### Cryptosporidiosis in cattle

There are four main *Cryptosporidium* species that infect cattle: *C. parvum*,* C. andersoni*,* C. bovis*,* and C. ryanae. Cryptosporidium parvum* is the main cause of cryptosporidiosis in cattle. Cryptosporidiosis in pre-weaned calves is almost always caused by *C. parvum*, resulting in clinical disease during the first weeks of their life. Pre-weaned calves under three weeks of age are highly susceptible to *C. parvum*. A meta-analysis reported high infection prevalence in calves and pre-weaned cattle, ranging between 27 and 37% [25], and a further meta-analysis focusing on *C. parvum* in dairy cattle reported a global prevalence of around 22% [26]. Clinical signs typically appear in calves from 3 to 4 days of age and range from mild to severe disease, which usually lasts for 1–2 weeks [2]. Clinically affected calves typically show profuse watery diarrhea, dehydration, reduced feed intake, and impaired growth, although asymptomatic infection also occurs. Fever is usually absent, and systemic signs are largely secondary to dehydration rather than direct invasion of epithelial cells, and the disease is usually self-limiting [2]. Mortality caused by cryptosporidiosis alone is generally low, but deaths can occur in cases of severe dehydration and often linked to poor management, or co-infections with, for example, rotavirus, coronavirus or enterotoxigenic *E. coli* [27]. Even when mortality is limited, cryptosporidiosis is associated with significant production losses due to delayed growth [4].

While *C. parvum* dominates in pre-weaned calves and is the main cause of cryptosporidiosis, other *Cryptosporidium* species are more frequently detected in older age groups. Molecular studies show that *C. bovis* and *C. ryanae* are commonly found in post-weaned calves and young stock, while *C. andersoni* predominates in adult cattle and infects the abomasum rather than the intestine [28, 29]. Although there tends to be an age-related distribution of the different *Cryptosporidium* species within cattle, this is not set in stone and co-infection of *C. parvum* and *C. bovis*, as well and *C. parvum* and *C. ryanae*, can be detected in pre-weaned calves [29]; shedding of *C. bovis* and *C. ryanae* can continue sporadically for many months [30] but is not usually associated with diarrhoea [31, 32]. Infection with *C. parvum* is also not just limited to pre-weaned calves, as this species of *Cryptosporidium* can also be found in older calves and adult cattle [23, 33]. In post-weaned calves and adult cattle, infection with *Cryptosporidium* is common but clinical disease is rare. Nevertheless, they can shed oocysts intermittently [28], thereby acting as reservoirs that contribute to environmental contamination and exposure to susceptible calves. Mortality associated with cryptosporidiosis in adult cattle is negligible, but infection of adult dairy cattle with *C. andersoni* has been linked to reduced growth and milk yields [34].

It should also be noted that there is a distinct global distribution of infection; whereas in Europe, *C. parvum* predominates in infections of calves, this is not true globally. In many sub-Saharan African countries, cattle, including calves, are generally infected with other species of *Cryptosporidium*, including *C. ubiquitum*,* C. andersoni*, and *C. bovis* [11]. This could be related to farm management (extensive farming predominates in these countries, and where *C. parvum* is reported, it is often associated with intensive farming), cattle breed, or other variables [11]. The symptoms of cryptosporidiosis, when disease is associated with these infections, tend to be relatively mild.

### Cryptosporidiosis in sheep and goats

Three different *Cryptosporidium* species are commonly detected in sheep and goats: *C. parvum*,* C. ubiquitum*,* and C. xiaoi* [35, 36], but which of these species is predominant in sheep varies geographically, as with cattle. Whereas *C. parvum* is most prevalent in Europe, *C. xiaoi* occurs most often in Oceania, Asia, and Africa, while in the Americas *C. ubiquitum* is most prevalent [35, 37]. In contrast to cattle, small ruminants do not tend to exhibit marked age‑related differences in the distribution of the three predominant *Cryptosporidium* species [38]. Disease is seen in neonatal lambs and goat kids and is usually associated with *C. parvum* infection. Cryptosporidiosis manifests by neonates becoming depressed, reluctant to nurse, and suffering from watery, yellow diarrhoea. Older animals can still become infected, but their infections tend not to be associated with diarrhoea or disease. This was demonstrated in a study, where 1-week-old and 6-week-old lambs with no prior exposure to *C. parvum* were infected with *C. parvum* oocysts; both groups became infected and shed large numbers of oocysts, but whereas the younger lambs developed symptoms such as anorexia, lethargy, and diarrhoea, evidence of clinical disease was negligible in older lambs [39]. *Cryptosporidium xiaoi* infection in lambs has also been associated with mild to severe diarrhoea and can also lead to mortality [3].

### Cryptosporidiosis in pigs

Porcine cryptosporidiosis has been documented globally with a prevalence of around 16% [40]. Although at least eight *Cryptosporidium* species have been detected in pig faeces, natural infections are dominated by the pig-adapted *C. suis* and *C. scrofarum*. Although both these species infect pigs of all ages, field studies show age-related patterns with *C. suis* being more common in pre-weaned piglets, whereas *C. scrofarum* predominates in weaned and older pigs [41, 42]. Zoonotic *C. parvum* occasionally infects pigs (about 2–3% globally) [40]. Other species are detected only sporadically, and their presence in faeces most likely reflects mechanical passage rather than true infections. In an experimental study it was demonstrated that pigs are not susceptible to rodent *Cryptosporidium* species, *C. muris* and *C. tyzzeri*, even when exposed to high doses (1 × 10^7^ oocysts) [43]. Other species such as *C. hominis* and *C. meleagridis* were shown experimentally to be able to infect gnotobiotic pigs, but natural infections have not been reported [44, 45].

Clinical outcomes vary widely and are likely to reflect infections of animals at different ages and with different *Cryptosporidium* species. Most porcine infections remain subclinical, but experimental infections with *C. parvum* have produced signs such as anorexia, lethargy, vomiting, and diarrhea [3]. Infections with *C. suis* or *C. scrofarum* are usually less severe than those induced by *C. parvum*. Co-infections can exacerbate disease; for example, rotavirus co-infection markedly increased illness and mortality in piglets experimentally infected with *C. suis*, while rotavirus-free piglets exhibited only mild signs [46]. Developmental stages occur throughout the intestine, producing lesions that may include villous atrophy and fusion, lamina propria infiltration, and occasional epithelial sloughing. All evaluated species cause comparable intestinal damage, although lesions associated with *C. parvum* tend to be more pronounced.

### Cryptosporidiosis in poultry

Cryptosporidiosis is a relevant disease of birds, including poultry, ratites, and gamebirds [3]. The most important *Cryptosporidium* species of birds include: *C. baileyi*,* C. meleagridis*,* C. galli*, *C. avium* and *C. ornithophilus*, and there are also several genotypes for which formal species names are lacking. These *Cryptosporidium* species and genotypes exhibit differences in host specificity, tissue predilection, and the clinical manifestations associated with infection. *Cryptosporidium baileyi* and *C. meleagridis* are more frequently reported and apparently cause the most severe clinical symptoms; they are therefore the focus here. According to [47], cryptosporidiosis is more severe in turkeys than chickens and is frequently fatal in quail.

*Cryptosporidium baileyi* is relatively unique in that its most characteristic presentation in poultry, especially broiler chickens and turkeys, is in the respiratory tract where it causes respiratory cryptosporidiosis, characterized by various symptoms including coughing, sneezing, dyspnea, nasal discharge, depression, lethargy, poor growth and increased mortality [48]. Ducks have also been reported to be infected, although with milder symptoms.

However, *C. baileyi* infections are not limited to the respiratory tracts of birds, with other sites also susceptible to infection including the intestinal tract and hind gut, the cloaca and bursa of Fabricius, and, more rarely, the ocular tissues and the kidneys [8]. While intestinal infections are often less severe than intestinal cryptosporidiosis in calves, the symptom spectrum is similar and includes diarrhoea, dehydration, and reduced weight gain. Cloacal and bursal infection causes inflammation and swelling, with impaired immune responses due to bursal damage, and renal cryptosporidiosis causes enlarged kidneys, polyuria, dehydration, and mortality in severe cases [48].

*Cryptosporidium meleagridis* most commonly infects turkeys (especially young poults) and chickens, but also game birds such as quail and pheasants, and occasionally ducks. Although experimental infections in chickens have indicated that *C. baileyi* may cause more severe infection than *C. meleagridis*, based on oocyst shedding [49], outbreaks of disease have occurred. In one outbreak on a game farm, morbidity among red-legged partridges (*Alectoris rufa*) was 60–70% in birds between 4 and 25 days, with mortality over 50% [50]. It is also worth noting that *C. meleagridis*, unlike most other species of *Cryptosporidium* infecting poultry, is zoonotic, and considered to be the third most common species of *Cryptosporidium* infecting humans globally [48, 51].

### Immune responses to *Cryptosporidium* infections in livestock

Immune responses to *Cryptosporidium* parasites have been studied in detail in mouse models but the responses to the parasite in livestock species are not that well understood [2]. Neonatal ruminants are of particular interest as they are born immunologically naïve due to the structure of the maternal placenta, which does not allow the transfer of antibodies, immune cells or cytokines *in utero* [52]; this may explain why neonatal ruminants are particularly sensitive to *C. parvum* infection. A review by Thomson and colleagues [2] describes the innate immune responses from epithelial cells, natural killer cells, γδ T cells, dendritic cells and macrophages against the parasite as well as the adaptive immune responses including CD4 + T cells, Th1 responses, Th2 responses, Th17 responses, CD8 + T cells and B cells but they conclude that a more in depth understanding of the pathogenesis and the immune responses in clinically relevant host species is required [2].

Longitudinal and experimental studies have shown that animals can be re-infected with *C. parvum* [28, 39]. The longitudinal study followed dairy calves for the first year of their lives and it was noted that all young calves on the farm became infected with a distinct *C. parvum* subtype and at six months of age some of the calves were shedding a different *C. parvum* subtype [28]. A follow-up experimental infection study demonstrated that lambs that had previously been infected with *C. parvum* could be re-infected when they were six weeks old with the same subtype [39]. The re-infection did not result in clinical symptoms, but the lambs were shedding significant numbers of oocysts [39]. These studies show that cattle and sheep do not develop sterile immunity, which prevents them from re-infection / shedding. Interestingly, the change in age-related susceptibility to disease cannot be explained completely by the development of an adaptive immune response because lambs that had been kept *C. parvum*-infection free for the first six weeks of their lives also became infected with *C. parvum* when challenged, although they did not develop symptomatic cryptosporidiosis [39].

Infection with *Cryptosporidium* induces both humoral and cell-mediated immune responses; however, protective immunity in neonates is limited due to the absence of endogenous antibodies and an immature cellular immune response at birth [4]. Consequently, serum antibody detection is not useful in neonatal calves, and susceptibility to infection is highest during the first one to two weeks of life [11].

While cellular immunity is considered central for long-term protection against *Cryptosporidium*, colostrum antibodies can contribute to early mucosal defence by limiting parasite attachment, invasion, and replication within the intestinal epithelium [4, 53]. Colostrum contains immunoglobulins (predominantly IgG1 in cattle), as well as a wide range of bioactive components, including cytokines, growth factors, antimicrobial peptides, leukocytes, and nonspecific immune mediators. Experimental and field data indicate that high titres of parasite-specific antibodies in colostrum are associated with reduced severity and duration of diarrhoea in neonatal calves [54, 55].

The importance of parasite-specific antibodies has been reinforced by the development and commercialisation of maternal vaccines targeting *C. parvum*. Vaccination of pregnant cows and heifers with a subunit vaccine containing the gp40 antigen results in significantly elevated levels of anti‑*Cryptosporidium* antibodies in colostrum compared with those induced by natural exposure alone (see section “VACCINES AGAINST CRYPTOSPORIDIOSIS” in this article; [54, 55]). Calves that ingest colostrum from vaccinated dams acquire passive immunity that has been shown to reduce the severity and duration of neonatal diarrhoea caused by *C. parvum*, and to reduce oocyst shedding, although shedding is not completely eliminated [54–56].

## Diagnostic tools

Current diagnosis of a *Cryptosporidium* infection is usually based on the detection of oocysts, antigens or DNA in faecal samples [57]. Occasionally, organisms can also be found in intestinal fluid, tissue samples, or biopsy specimens [58]. There is no gold-standard method for diagnosis, and the approach used is often based on the resources available within the specific setting, whether it is important to know the species or subtype of *Cryptosporidium* causing the infection, or whether on-farm or near-pen testing is required. There are various methods to choose from for each of these scenarios, including microscopy, immunological and molecular based assays, and there are various commercially available kits. Differentiating Infected from Vaccinated Animals (DIVA) tests are neither required nor available for *Cryptosporidium*.

### Sample treatment and preparation

Ideally, specimens should be collected for the primary diagnosis of *Cryptosporidium* during the acute infection and stored short-term at 4 °C to retain optimal morphology. However, if chilled storage is not available then preservatives can be added but must be selected carefully so that downstream assays that may be required are not affected. Examples of preservatives used include: potassium dichromate, 10% aqueous formalin, merthiolate-iodine-formaldehyde, sodium acetate-acetic acid-formalin and polyvinyl alcohol [58]. While these preservatives are mostly compatible with microscopy or immunological methods, many are inhibitory to PCR or other molecular assays. If PCR testing is required, preserving specimens in 90% ethyl alcohol is also suitable.

Pre-processing methods may also be required prior to testing. This may be to remove preservatives, to concentrate the oocysts into a small volume if sampling was performed during a late infection or the stool sample is very liquid, or to remove faecal components that could be problematic during testing, such as high levels of fat or mucus, or fibrous material [58]. While concentration is compatible with microscopic and molecular methods, it is generally not appropriate for immunological assays where the target soluble antigens may be lost. Methods for concentrating oocysts in faeces include sedimentation or flotation, using saturated salt, sucrose or zinc sulphate solutions [58]. An acid flocculation concentration method has also been described that allows relatively large (50 g) amounts of faeces to be concentrated, thereby increasing the sensitivity of detection when few oocysts are present [59].

### Microscopy-based tools

Detection of oocysts in faeces by microscopy has been the cornerstone of *Cryptosporidium* diagnostics for several decades [60]. Primarily this has been through the examination of faecal smears stained with tinctorial stains such as modified Ziehl-Neelsen (mZN) or auramine phenol. The main advantages of these methods are the low cost and the relatively basic equipment that is commonly found in most laboratories globally, particularly for mZN, which only requires a simple light microscope. The main disadvantages of using these methods are that the sensitivity can be low, experienced technicians are required to examine the samples, and *Cryptosporidium*-like artefacts can cause equivocal results. The use of immunofluorescence microscopy (IFM), which utilizes fluorescently labelled anti-*Cryptosporidium* oocyst wall antibodies for staining oocysts in faecal smears, is also common in some settings, bringing improved sensitivity and specificity, but requiring additional resources to meet the increased cost of the reagents and a fluorescence microscope [58].

In some circumstances, usually post-mortem, haematoxylin and eosin staining can be used on tissue samples or biopsies for histological diagnosis of *Cryptosporidium* by detecting the endogenous stages of the parasite and the pathology consistent with cryptosporidiosis [58].

### Immunological tests

The detection of *Cryptosporidium* antigens in faecal specimens has become a popular diagnostic approach in recent years using commercially available methods such as enzyme immunoassays (EIA) or immunochromatographic lateral flow (ICLF) tests. As with all diagnostic assays there are advantages and disadvantages that may dictate which method is most appropriate for a particular setting. EIAs provide improved sensitivity and specificity, with less technical expertise needed, over simple microscopy methods such as mZN staining, and are additionally amenable to high-throughput testing both manually and through automation, but come at a higher financial cost [58]. The use of ICLF tests removes the requirement of skilled technicians and, due to their portability and rapid results, are commonly used for on-farm and near-pen testing [60], allowing differential diagnosis of *Cryptosporidium*, rotavirus, coronavirus, and enterotoxigenic *E. coli*; however, they are more expensive and their sensitivity is lower [58].

Most of these commercial kits have been developed from *C. parvum*-derived antibodies and therefore the presence of antigens from other species may not be detected as readily [61]. However, as *C. parvum* is usually the main pathogenic *Cryptosporidium* species in livestock, this may not be problematic.

### Molecular diagnostic tools

Molecular diagnostic tests for *Cryptosporidium* are nowadays widely used for human infections, particularly in high-income countries, often as part of a gastrointestinal multi-pathogen screening assay. However, their use in animal testing is currently relatively rare. While molecular assays generally have high sensitivity and specificity, this can be dependent on the infecting *Cryptosporidium* species and compatibility with the target locus and primer set included. However, as with immunological tests, most commercial kits have been developed predominantly for diagnosis of infection with *C. parvum* and closely related species. An important limitation to using molecular diagnostic tools is the expense, as the cost per test can be relatively high; in addition, the requirement for expensive and specialized equipment can prohibit their use in low-resource settings [62]. In addition to PCR-based molecular assays, there are a few reports of loop-mediated isothermal amplification (LAMP) being used to detect *Cryptosporidium* in animals [63, 64]. LAMP is an attractive molecular technology due to its low cost, being isothermal (no need for an expensive thermal cycler), and high sensitivity. However, the high sensitivity and the use of multiple primers increase the risk of primer‑dimer formation and other non‑specific amplification events, which can lead to false positive results; cross-contamination is also recognised as a serious issue with LAMP [65].

Most of the currently available commercial molecular diagnostic tests do not identify the *Cryptosporidium* species present but rather utilize real-time PCR technology to indicate whether the sample is positive for the genus. Additionally, there are many non-commercial assays that have been published targeting various loci that utilize both conventional and real-time PCR. Conventional PCR methods often use a nested PCR approach to further increase sensitivity and specificity. The most common target for PCR-based detection of *Cryptosporidium* is the small subunit rRNA (*ssu* rRNA) gene as this locus has low levels of intraspecific variation and therefore can be used to detect all known species by sequencing [66]. Moreover, it is a multi-copy locus in the genome, and this increases the test sensitivity. Other loci that have been used for detection of *Cryptosporidium* in animal samples are the *Cryptosporidium* oocyst wall protein (COWP), actin, thrombospondin-related adhesive protein (TRAP-C2), and 70 kDa heat shock protein (*hsp70*) genes [66].

### Molecular typing tools for epidemiological or outbreak investigations

Identification of the infecting *Cryptosporidium* species becomes particularly important during epidemiological surveillance and outbreak investigations involving zoonotic transmission. In these circumstances, species will usually be characterised using PCR combined with amplification product sequencing or, less frequently, restriction fragment length polymorphism (RFLP). While sequencing remains the reference approach for comprehensive species identification, species-specific real-time PCRs are available, although their extent of validation is variable, these do not cover the full diversity of species and genotypes, and most are designed for use with human clinical samples. Although often used in a research setting, these methods are usually only performed at specialist or reference laboratories.

In addition to species identification, subtyping can provide crucial data on the epidemiological linkage between samples. For many years the method of choice for subtyping *Cryptosporidium* species has been by DNA sequence analysis of a hypervariable 60 kDa surface glycoprotein gene (*gp60*). This single locus target has been described in many species and often include several variable features that have been used to determine the subtype [67]. Depending upon the species of *Cryptosporidium* these may include a microsatellite region, minisatellite repeats, and various polymorphisms in the sequence throughout the gene. However, due to the obligatory sexual stage of the parasite’s life cycle where recombination is known to occur, a multilocus approach, with markers throughout the genome, is more appropriate [68].

Several multilocus typing methods have been published and used to subtype isolates from a few *Cryptosporidium* species infecting animals [69–71]. Due to its pathogenic importance to both animals and humans, *C. parvum* has been the target of many of these methods [72–75]. Two approaches have been used for multilocus typing of *C. parvum*: sequencing (MLST) and fragment sizing using multiple loci variable number of tandem repeat analysis (VNTR), often abbreviated to MLVA. Both approaches have shown much greater discriminatory power than single-locus *gp60* subtyping and are useful in population structure studies [76], epidemiological surveillance, and investigation of animal-related outbreaks [77, 78]. The advantage of using MLST is that every sequence variation is available, but disadvantages are the high cost and the time that is required to generate and analyse the data; in addition, mixed infections can still be difficult to identify. MLVA, however, is rapid and cheaper, enabling contemporary investigation, and as the peaks represent the length of each allele, mixed infections can be easily seen by the presence of multiple peaks. The limitation is that this approach does not detect every sequence variation that may be present. More recently, whole genome sequencing (WGS) has been used to compare *C. parvum* isolates from various hosts and geographic origin, allowing a better understanding of the population structure and molecular evolution of this parasite [79, 80]. However, WGS is costly, time consuming and requires either purification of oocysts before DNA extraction or targeted capturing and sequencing of the small fraction of Cryptosporidium DNA present in faecal samples [81]. WGS also requires bioinformatic competence, which has limited its application to a few specialist laboratories. Interestingly, from a practical point of view, comparison of MLVA profiling with WGS data demonstrates concordant epidemiological clustering, although WGS provided more insight into mixed infections [82].

### Gaps and future needs

Currently, there are no diagnostic kits or methods described and validated by International (WOAH), European (EU) or National Standards. Specialist reference laboratories will provide most *Cryptosporidium* diagnostic methods under ISO accreditation and commercially available kits are usually also accredited in some form. In 2024, WOAH removed the chapter on cryptosporidiosis from the *Manual of Diagnostic Tests and Vaccines for Terrestrial Animals*. Although the chapter updated in 2022 remains available in the online archive, it is no longer revised.

There are advantages and disadvantages associated with each of the wide variety of diagnostic tools available for the detection of *Cryptosporidium* in livestock. Due to the importance of cryptosporidiosis, both clinically and economically in an agricultural setting, it should always be included in the standard testing of neonates for diarrhoeal diseases [83]. The choice of which method to use is dependent on numerous factors including the resources available and the aim of the diagnosis (e.g. surveillance, cause of clinical illness, outbreak support). There is currently a lack of evaluation of these diagnostic assays for each diagnostic target: clinical, subclinical, or epidemiological diagnosis.

Although molecular-based platforms for rapid detection are established and commonly used within the human clinical setting, they are not widely used in veterinary diagnostics. Additionally, the diagnostic tests that are currently commercially available only detect the presence of the genus *Cryptosporidium*. There may be some commercial potential for molecular assays that are designed to differentiate between species and possibly subtypes, as not all *Cryptosporidium* species infecting animals represent the same zoonotic potential or are associated with the same extent or severity of disease. Although this may not be a requirement in everyday veterinary practice, such a tool could be of value in outbreak investigations and tracking of infection transmission.

Some opportunities exist for development of diagnostic tools for *Cryptosporidium* infections in animal hosts that may be beneficial. Although cheap, reliable, on-site diagnostic kits for veterinary use are available, currently none are specific for *C. parvum* which could be of value, given its clinical importance in both veterinary and human infections. Further development of sensitive and cheap methods for diagnosing early infection would also be of value, as well as those that can monitor oocyst shedding accurately. There are also no validation data of commonly used ICLF tests for different host species (such as poultry and pigs) available. Although pen-side Point of Care (POC) tests are available in some countries, national regulatory frameworks governing animal health products vary widely. Thus, these tests are available in many European Countries, but not, for example in the USA. The development and challenges of improved POC tests for *Cryptosporidium* have been described, including potential benefits from using technology such as CRISPR and LAMP for POC detection and genotyping [65].

WGS of relevant species and genotypes has enhanced our knowledge of *Cryptosporidium* biology and enabled us to develop new molecular diagnostic tools. However, it is currently difficult, laborious, and costly to generate WGS data from subclinical or asymptomatic infections, while this may be important to understand drivers of virulence. Genome-targeted enrichment technology shows potential in alleviating this but is still under development [84]. Additionally, fully sequenced and annotated genomes are lacking for many species and genotypes.

## Vaccines against cryptosporidiosis

Vaccination has long been considered a desirable control option for bovine cryptosporidiosis because *C. parvum* is highly transmissible, affects calves during a very narrow early-life window, and its environmentally robust oocysts make elimination by hygiene alone unrealistic [2, 11]. In bovines, the key practical constraint is that clinical disease typically occurs within the first 1–3 weeks of life, leaving insufficient time for a conventional “direct-to-calf” vaccination strategy to induce protective active immunity prior to exposure [2]. Consequently, the leading veterinary approach has been maternal immunisation, designed to raise *Cryptosporidium*-specific antibodies in colostrum and thereby provide passive protection immediately after birth [54, 55, 85, 86].

### Current status of vaccines for veterinary and human cryptosporidiosis

For many decades after the discovery of the importance of cryptosporidiosis as a disease of both people and livestock, licensed vaccines against cryptosporidiosis were unavailable for either group, reflecting major biological and technical barriers including incomplete understanding of correlates of protection, challenges in antigen discovery, and limited tractable culture and in vivo systems [2, 87]. However, over the last few years, bovine maternal vaccines based on *C. parvum* surface antigens have reached the market in some regions, representing a substantial change in the control toolbox. In parallel, advances in experimental models — such as a genetically tractable natural mouse model (*C. tyzzeri*) that supports vaccine testing—are providing new opportunities to accelerate rational vaccine design for both veterinary and human applications [87].

### Antigen selection and mechanistic rationale: the gp40/gp900 glycopeptide epitope

A central development in bovine vaccine design has been the identification and functional validation of epitopes on mucin-like glycoproteins involved in host-cell invasion. Studies of a protective monoclonal antibody (4E9), recognising a glycopeptide epitope present on *C. parvum* gp40 and gp900. Bhalchandra et al. [88] showed that this antibody can ameliorate disease in vivo and demonstrated epitope localisation on invasive and intracellular stages. These findings provide strong biological support for gp40-focused maternal vaccination: antibodies induced in the dam can bind parasite stages relevant to early intestinal infection and can be transferred via colostrum to protect neonates during the peak-risk period [54, 55, 88].

### Commercial maternal subunit vaccination in cattle: evidence base

Experimental challenge evidence: Timmermans et al. [54] evaluated recombinant *C. parvum* gp40 vaccination in pregnant cows, demonstrating markedly increased anti-gp40 antibody titres in dams, transfer of these antibodies to calves via colostrum, and, under controlled challenge, significant improvements in calf health. These improvements included reduced incidence and duration of severe diarrhoea and increased weight gain in suckling calves. These data substantiate the mechanistic concept that immune colostrum induced by maternal vaccination can meaningfully mitigate clinical cryptosporidiosis in neonates.

Field/clinical study evidence: Reijnders et al. [55] reported two clinical studies conducted during the marketing authorisation process, noting that natural challenge levels were not sufficiently high to show significant reductions in most clinical signs, but that trends favoured vaccination and that diarrhoea duration in dairy calves was significantly shorter in the vaccination group; antibody levels against gp40 were significantly higher in colostrum and calf serum in vaccinated groups, confirming successful passive immunisation. Together, these studies indicate that maternal gp40 vaccination can (i) reliably raise specific antibodies and (ii) reduce clinical impact under some field conditions, while also highlighting the importance of infection pressure and study power for demonstrating field-level clinical endpoints [55].

### Expected effects and limitations: disease reduction rather than sterile immunity

Even with successful maternal vaccination, sterile immunity is not expected for *Cryptosporidium*, and although oocyst shedding may be reduced, it may not be eliminated entirely [2, 55]. This distinction is important for control strategies: vaccines that reduce diarrhoea severity and duration can improve welfare and productivity, but may not fully prevent transmission or zoonotic risk, particularly in high-contamination environments [2, 55].

A further practical limitation of maternal vaccination is its dependence on colostrum and transition milk management (timely intake, sufficient volume and quality), because the protective mechanism relies on passive transfer [54, 55]. This link reinforces the need to integrate vaccination with robust neonatal management (see Section on “OTHER MEASURES AGAINST ON-FARM TRANSMISSION OF CRYPTOSPORIDIOSIS – Colostrum management” in this article).

### Evaluation endpoints and harmonisation needs

A recurring challenge in cryptosporidiosis vaccine development is defining and standardising efficacy endpoints that reflect both animal health and transmission impact. Endpoints used across studies include diarrhoea incidence, duration, and severity, treatment requirements, weight gain, and measures of oocyst shedding [54, 55]. The lack of agreed evaluation criteria, such as the necessary reduction in oocyst excretion versus clinical benefit, remains an important gap for both vaccination and other treatment strategies. From a control perspective, clinical endpoints are critical for producers (welfare, labour, antimicrobial use), while shedding endpoints are essential for modelling environmental contamination and One Health risks; both are needed to compare products and optimise integrated control programs [2, 55].

### Pipeline approaches: beyond maternal subunits

Although maternal gp40 subunit vaccination is currently the most advanced veterinary strategy against bovine cryptosporidiosis, a broad set of approaches remains relevant:

(i) Additional subunit antigens and reverse vaccinology. Early vaccine discovery investigations for *C. parvum* investigated diverse antigens, including Cp15, Cp23, Cp7, profilin, and others. Notably, Cp15-based strategies, evaluated by Manque et al. [89] via reverse vaccinology and heterologous prime–boost regimens, elicited both humoral and cellular immune responses. Their work specifically covered Cp15, profilin, and apyrase. Cp23 is recognised as an immunodominant surface antigen, with partial protection demonstrated in passive and active immunisation models, including recombinant protein and DNA vaccines [90, 91].
(ii) Live-attenuated or genetically attenuated parasites. The *C. tyzzeri* mouse model has demonstrated that vaccination with attenuated parasites can provide protection comparable to prior infection, and that both host and parasite can be genetically manipulated; important attributes for systematic testing of immune mechanisms and vaccine strategies [87]. While this is not directly a cattle-product pathway yet, it nevertheless provides a conceptual and experimental foundation for next-generation vaccine design.
(iii) Human vaccine considerations. Cryptosporidiosis is a major paediatric diarrhoeal disease globally, and immune protection is complex and likely requires durable mucosal immunity. Progress in experimental models and antigen definition, including epitope-focused work, are directly relevant to human vaccine ambitions, but no broadly licensed human vaccine exists at present [2, 87, 88].

### Regional availability note

For completeness, it is worth noting that a US cattle cryptosporidiosis vaccine (maternal, colostrum-mediated protection) has been described in industry communications as becoming available in 2025 [92]. However, such announcements are not a substitute for peer-reviewed efficacy and safety datasets and should be interpreted accordingly.

### Knowledge gaps and research priorities specific to vaccines

Priority gaps in vaccine development against cryptosporidiosis include: (i) defining the magnitude of reduction in oocyst shedding that is necessary to have a meaningful impact in reducing transmission; (ii) establishing harmonised efficacy endpoints across studies; (iii) clarifying correlates of protection (humoral versus cellular and mucosal components); and (iv) extending vaccine approaches to other livestock hosts (e.g., sheep, goats, deer) and, potentially, to other *Cryptosporidium* species of veterinary or zoonotic relevance. In addition, continued work is needed to understand whether antigenic diversity (e.g., subtype variation) can influence field effectiveness and whether multivalent antigen strategies could further improve breadth and robustness of protection [88].

## Other measures against on-farm transmission of cryptosporidiosis

### General farm management and hygiene

Cryptosporidiosis control in livestock is essential for maintaining good animal welfare, protecting public health, and reducing the economic losses associated with the disease. As infected calves shed millions of oocysts and only a small number are required to establish infection [3], complete elimination of *Cryptosporidium* from affected farms is unlikely, but disease impact can be significantly reduced through integrated management [93]. An effective control strategy at the farm level must focus on reducing environmental contamination, limiting calf exposure, and interrupting transmission pathways. This requires a combination of strict hygiene, targeted biosecurity, environmental control, and appropriate husbandry practices (including good colostrum management; see colostrum management sub-section below) [93].

*Cryptosporidium* oocysts are resistant to many disinfectants, which means that hygiene programmes must emphasize physical removal of organic material, followed by using disinfectants that are effective against *Cryptosporidium* to reduce the environmental oocyst load (see Disinfectants and inactivation of oocysts sub-section below).

However, even if the majority of oocysts are inactivated, infection may still occur, and pens can be rapidly re‑contaminated when animals start shedding oocysts. Therefore, hygiene must be continuous and rigorous, and its successful implementation plays a critical role in the successful rearing of young ruminants.

Many different management practices have been suggested to reduce the impact of *Cryptosporidium* infection on their farms, and these are highlighted in special publications for farmers (e.g [94]), . However, these management options, despite being logical, seem to be often based on limited, or even contradictory, research findings. A recent systematic review of scientific literature evaluated many different management practices for bovine cryptosporidiosis and found no clear support that the following practices had reliably protective effects: isolation of sick calves from healthy calves; different types of bedding; different protocols for cleaning feeding utensils; reduced contact with other calves; individual calf pens; and feeding nutritional supplements [93]. The same systematic review found only limited evidence that other factors had some protective effects. These factors included; lower stock density; slatted flooring; increased depth of bedding; frequent removal of faecal matter from pens; prevention of co-infection with other pathogens that can cause diarrhoea; and calving in cooler, less humid months. Consistent evidence of protective effects was found for just two factors: smaller herd sizes and concrete flooring [93]. The review concluded that, in general, the scientific evidence to support most specific and recommended farm managements practices to control cryptosporidiosis is either entirely absent or of poor quality, and that there is a need for better data obtained from more high-quality studies investigating specific control practices to make solid evidence-based recommendations [93].

Although it seems that many recommended farm management practices may have poor support from studies, good colostrum management is a cornerstone of cryptosporidiosis control. However, there are some conflicting findings with regard to other feeding practices. For example, an increased risk of cryptosporidiosis has been observed when milk replacer is used, with some studies reporting increased shedding [95], while others report decreased shedding [96]. This could be related to other factors associated with farms included in those studies. It has also been suggested that providing starter grain in the first week of life may increase the risk of cryptosporidiosis, possibly due to abrupt changes in gut microflora.

Cryptosporidiosis is a zoonotic disease, and control on farms has direct implications for public health. Collaboration between veterinarians and farm managers within a One Health approach is essential for implementing integrated management practices to help with prevention and control of cryptosporidiosis, thereby reducing the impact of infection on calf health and farm productivity and minimizing potential transmission to humans. However, it is also clear that many current recommendations are not well grounded in good-quality studies providing clear data.

### Colostrum management

Adequate colostrum management is essential for neonatal health in ruminants and represents one of the most important indirect measures to mitigate the transmission and impact of *C. parvum* infection in calves. As cryptosporidiosis primarily affects neonates during the first weeks of life, when the adaptive immune system is immature, protection relies largely on passive immunity and nonspecific immune factors transferred via colostrum [55, 97, 98].

Passive transfer of immunity via colostrum therefore plays a critical role in reducing disease severity and supporting early host defence [4, 97].

Field and experimental studies have demonstrated that passive immunity begins immediately after ingestion of colostrum and is most effective when calves receive sufficient quantities of high-quality colostrum shortly after birth. Key features of successful colostrum management are speed of delivery, amount, quality, and cleanliness. Protection is transient, typically extending through the period of highest susceptibility during the first two to four weeks of life [11, 56, 98]. These observations underscore the dual importance of both colostrum quality (including specific antibody content) and colostrum management practices.

From a transmission perspective, colostrum does not prevent infection *per se* but can substantially reduce clinical disease, thereby indirectly influencing environmental contamination and onward transmission. Calves with milder or shorter diarrhoeal episodes are likely to shed fewer oocysts overall and for a shorter duration, reducing contamination of housing, bedding, and fomites [4, 11]. Given the extremely low infectious dose of *C. parvum* and the high environmental resistance of oocysts, even partial reductions in shedding may have epidemiological relevance at herd level [11, 93].

Failure of passive transfer, due to delayed feeding, insufficient volume, poor-quality colostrum, or bacterial contamination, has consistently been identified as a risk factor for severe neonatal diarrhoea, including cryptosporidiosis [11, 97]. In farm situations where cryptosporidiosis is endemic, inadequate colostrum intake can exacerbate the impact of environmental exposure, while optimal colostrum management can buffer calves against high infection pressure [4, 97].

### Disinfectants and inactivation of oocysts

The main challenge for disinfecting farm environments is that the oocyst wall of *Cryptosporidium* parasites is very tough and resistant against many available disinfectants. The *Cryptosporidium* oocyst wall has two main layers. The outer oocyst wall layer consists of acid-fast lipids, which are predicted to give the oocysts a waxy coating that offers protection against many disinfectants commonly used on farms, while the inner layer provides structural integrity [2, 99]. The oocyst wall prevents many disinfectants from penetrating the oocysts and inactivating the sporozoites inside, which is a challenge when the parasite has such a low infectious dose.

Research on the efficacy of different disinfectants and chemical compounds in the inactivation of *C. parvum* oocysts has been extensively reviewed in two publications from some years back [100, 101]. There is general agreement between studies that higher temperatures (45 °C for 20 min; 50–55 °C for 5 min [102, 103], desiccation (air drying for 4 h [9]) and ultraviolet radiation (low pressure UV light at 120–8748 mJ/cm^2^ in water [104, 105]), from both artificial light and sunlight, can inactivate *Cryptosporidium* oocysts [101]. Many studies on disinfectant efficacy have been conducted in clean (laboratory) environments, where the effect of faeces and other farm substrates have not been considered. The review by Fayer [101] also highlighted that temperatures at which disinfectants are used may affect their efficacy, where higher temperatures often increase the inactivation of oocysts, probably due to increasing oocyst wall permeability and therefore providing access of the disinfectant to the sporozoites.

The following chemical compounds have been assessed for their potential to inactivate oocysts and therefore to be suitable active ingredients for disinfectants for inactivation of *Cryptosporidium* oocysts.

#### Hydrogen peroxide

The use of hydrogen peroxide as a disinfectant for *Cryptosporidium* has been reviewed by both Casemore & Watkins [100] and Fayer [101]. Studies have shown that a 3% hydrogen peroxide solution is able to inactivate oocysts in 5 min [102]. Another benefit, in addition to the short contact time, is that hydrogen peroxide breaks down into water leaving no toxic residues. However, a disadvantage is that hydrogen peroxide reacts with organic material, which reduces its effectiveness against oocysts in the farm environment [106].

#### Ammonia

As a disinfectant for *Cryptosporidium* oocysts, ammonia has been shown to be similarly effective as hydrogen peroxide at various concentrations, with contact times ranging from 30 min to 24 h [101]. An ammonia-based commercial disinfectant was shown to result in a 97% reduction in oocyst viability as measured by excystation, at a concentration of 5% with a contact time of 30 min at 22 °C [103]. However, this product has been withdrawn from the European market.

#### Chlorine and bleach (sodium hypochlorite)

It is well documented that chlorination of drinking water does not inactivate *Cryptosporidium* oocysts as the necessary concentrations for inactivation needs to be significantly higher than used for drinking water (> 80 mg/L) with long contact times of up to 24 h [100, 101]. However, concentrations from 0.07 to 4.03 mg/L of chlorine dioxide with contact times from 15 to 90 min have resulted in 90–97% reductions in oocyst survival [101]. A 6% bleach solution, consisting of sodium hypochlorite, which releases free chlorine, did not inactivate *Cryptosporidium* oocysts with a 33-minute contact time [107]. 

#### Other compounds:

Other compounds that have been tested and resulted in some inactivation of *Cryptosporidium* oocysts include: monochloramine, iodine, formaldehyde, formalin, bromomethane gas, ethylene oxide, peracetic acid, potassium dichromate, potassium permanganate and sodium hydroxide [101]. Some of these compounds have been used in combinations with either hydrogen peroxide or ammonia in commercial disinfectants that have been shown to be effective at inactivation of *Cryptosporidium* oocysts [108].

## Conclusion

In conclusion, the advice to farmers is to clean (remove as much organic material as possible), dry (to avoid further dilution of the disinfectants) and then disinfect areas and equipment. An appropriate disinfectant with known provenance for inactivation of *Cryptosporidium* oocysts should be used and the guidance on contact time and working concentration of the disinfectant should be followed.

### Slurry and manure management

Information on slurry and manure management practices that will help to reduce environmental contamination with viable *Cryptosporidium* oocysts is limited. Spreading slurry and manure onto land is a significant source of environmental contamination and it is estimated that globally each year 3.2 × 10^23^ oocysts are applied to the environment via manure [109]. To reduce the risk from these oocysts, it is essential that slurry is well fermented and that manure is well composted before it is applied to fields as fertiliser.

#### Slurry management

Jenkins et al. [110] investigated the effect of ammonia, found in slurry and cattle urine, on *Cryptosporidium* oocyst survival at two different temperatures, 4 °C and 24 °C. They found that higher temperatures resulted in faster oocyst inactivation and estimated that all oocysts die off within 8.2 days in slurry with 0.06 M ammonia concentrations at 24 °C, but at 4 °C it was necessary for exposure for 55 days [110]. This study also reported that the ammonia concentration in slurry increases over time, and that this increase could inactivate oocysts even at lower temperatures. A mathematical model that was developed to predict the changes in ammonia levels at different levels within slurry storage tanks showed that ammonia is lost from slurry by volatilisation, but that this only affects the top 5–10 cm of slurry [111]. The model also predicts that the high ammonia levels in the slurry will effectively inactivate *Cryptosporidium* oocysts, except within the top few centimetres where the ammonia levels may be too low. Ruxton [111] suggested using management techniques that affect surface ammonia concentrations, such as occasional stirring or crusting of the slurry, to achieve complete inactivation of oocysts present within the slurry.

#### Manure management

*C. parvum* oocysts have been demonstrated to survive in faeces for prolonged periods [9], with up to 40% of oocysts remaining viable after being incubated in faeces for 176 days at ambient winter temperatures in Scotland. This raises the question of whether oocysts can survive in farm manure. Thapaliya et al. [112] used manure containing *Cryptosporidium* oocysts in temperature fluctuation chambers, which mimicked daily temperature variations set for US winter or summer ranges. They confirmed that *Cryptosporidium* oocyst viability in manure at winter temperatures (cycling between 1 and 18 °C, at a relative humidity of 50–90%) was only slightly reduced after 30 days, from 100% to about 91%. Using summer temperatures (cycling between 21 and 42 °C, at a relative humidity of 34–96%), resulted in a more pronounced drop in viability after 30 days, from 100% to about 60%. The authors speculated that the presence of ammonia in the manure negatively impacted oocyst survival. In addition to external temperatures, manure also generates heat during the composting process, which increases oocyst inactivation. However, this effect may be reduced at the bottom or the edges of the manure heap (midden), where generated heat is dissipated. Insulating the midden may help to allow the heat to build up and spread more evenly throughout the entire midden to inactivate oocysts. Alternatively, turning the midden in the early stages of composting, when most of the heat is generated, may increase inactivation of oocysts in all parts of the midden. A further risk is that rain may result in oocysts leaching out of the midden into the environment before inactivation; covering the midden could also reduce leaching of oocysts.

### Biosecurity

Sources of *Cryptosporidium* introduction into livestock farms include contaminated water (e.g., private supplies or surface water such as streams), the introduction of infected animals, farm visitors and associated vehicles, and wildlife reservoirs. The risk associated with contaminated water, particularly when used for reconstituting milk replacer, can be mitigated by using treated public water supplies or by installing ultraviolet (UV) irradiation systems to inactivate the oocysts [104, 105]. Where streams traverse fields housing neonatal ruminants, physical exclusion (e.g., fencing) can reduce exposure to oocysts transported from upstream sources. The introduction of new animals may facilitate the entry of more virulent *Cryptosporidium* isolates; while quarantine for several weeks can reduce this risk, it may not eliminate it entirely. Similarly, farm visitors and their vehicles can act as mechanical vectors; therefore, access to neonatal animals should be minimised, and, where contact is necessary, appropriate biosecurity measures (e.g., use of clean, disinfected protective clothing and footwear) should be applied. Wildlife species, including deer, geese, pheasants, and rabbits, have also been implicated as potential sources of infection [106, 113, 114] and should be kept away from neonatal livestock whenever possible.

Many subtypes of *C. parvum* are zoonotic, and livestock farming has been associated with human cryptosporidiosis through both direct and indirect transmission pathways [115]. Direct transmission commonly occurs during farm visits, particularly involving students or members of the public during lambing and calving seasons, when environmental contamination with oocysts is elevated. Such interactions, especially those involving direct contact with neonatal animals, have been well documented as sources of infection [115]. In the United Kingdom, 74 outbreaks linked to direct animal contact were reported between 2009 and 2017 [115], and in a survey of veterinary students in UK and Ireland, 15% reported acquiring cryptosporidiosis [116]. These outbreaks exhibit seasonal patterns, with peaks in spring and autumn, corresponding to spring lambing and calving and autumn calving periods. The risk of direct transmission can be reduced through minimising animal contact, implementing effective hand-washing practices, avoiding consumption of food and drink in animal areas, and preventing contamination of clothing and footwear.

Indirect transmission pathways include environmental contamination via farm runoff, contaminated fresh produce, and dairy products [115]. Runoff following land application of manure has been associated with increased incidence of human cryptosporidiosis [117]. Appropriate composting of manure and fermenting of slurry, as described in the sections above, prior to land application can reduce this risk. Contamination of vegetables and fruits may occur through the application of manure or slurry as fertiliser, or during post-harvest processing when contaminated water is used. This risk can be mitigated by using clean water in production systems and by thorough washing of produce before consumption. Milk and dairy products represent additional transmission routes, particularly when unpasteurised milk or dairy products are consumed or pasteurisation is inadequate [115]. Proper pasteurisation effectively eliminates this risk. Overall, enhanced dissemination of evidence-based guidance to farmers is required to minimise the release and transmission of *Cryptosporidium* from agricultural settings.

### Limitations and knowledge gaps regarding farm management measures for control of cryptosporidiosis

#### Colostrum

Despite the recognised importance of colostrum, several gaps remain. The specific immune components within colostrum that are most critical for protection against *Cryptosporidium* have not been fully elucidated. While antibodies against surface antigens, such as gp40, play a role, the contribution of other humoral and cellular factors, as well as interactions with the developing gut microbiome, requires further investigation [4, 53]. There is also a lack of data on the effectiveness of pooled colostrum, colostrum replacers, or supplementation strategies with respect to *Cryptosporidium* specific immunity. Understanding how colostrum-derived immunity interacts with natural exposure to build longer-term resistance remains an important research priority [4].

#### Disinfection

A major knowledge gap for the effectiveness of disinfectants is the lack of good comparable data for different chemical compounds or products. Most published studies use different compound concentrations, oocyst numbers, oocyst sources, oocyst ages, oocyst storage conditions, compound exposure times, temperature ranges and different viability assays. Variations in protocol conditions will lead to differences in results, even when the same compound is being tested. Therefore, there is a need to develop standardised protocols that allow the screening of different compounds or disinfectants for the inactivation of *Cryptosporidium* oocysts, which will allow for clear comparisons in the efficacies of different disinfectants.

#### On-farm management of slurry and manure

A review by King & Monis [118] highlighted that there is a need to evaluate the effect on oocyst survival when slurry is applied to fields used for grazing, where there are *Cryptosporidium* oocysts already present on the ground or in the soil.

Better scientific information, based on experimental studies, is needed about the inactivation dynamics of *Cryptosporidium* oocysts within a midden.

#### Biosecurity

A major limitation in farm biosecurity is the insufficient understanding of why farmers do not adopt existing biosecurity measures to protect livestock from cryptosporidiosis and to prevent the dissemination of *Cryptosporidium* oocysts beyond farm boundaries. Identification of barriers to uptake of effective measures within the farming community would be of value.

## Treatment of cryptosporidiosis – currently available and future needs

Pharmaceutical treatments of, and prophylaxis against, cryptosporidiosis in livestock, and in people, are relatively limited. One of the most important treatments is supportive care, such as oral electrolyte therapy, intravenous fluids in severe cases, nutritional support, and warmth and nursing care [119]. This supportive therapy is essential as dehydration and loss of electrolytes are the main causes of death in severe cryptosporidiosis, and use of bicarbonate-rich fluids along with potassium supplementation may also be of value [120]. Most cases of cryptosporidiosis are self-limiting. As development of immunity should be able to overcome the infection within a period of days or weeks, supportive therapy until the immune response is effective may be sufficient.

The pharmaceutical compounds available are only licensed for use in ruminants prophylactically or at the beginning of diarrhoea but not in animals that are severely affected. Their approval is also limited and not granted globally, so access is geographically restricted. Although other treatments are on the horizon, along with supportive therapies, there is no indication of new treatments or therapies reaching the market soon, indicating a control gap.

### Currently available treatments for livestock

There are two main pharmaceutical treatments available for cryptosporidiosis in livestock, the synthetic quinazolinone, halofuginone, usually supplied as halofuginone lactate, and paromomycin, an aminoglycoside antibiotic. However, these are not available in all countries; for example, neither of these are registered for use in food animals in USA. In Europe, the European Medicines Agency (EMA), recommends the use of paromomycin for calves, lambs, and goat kids [121–123], with subsequent withdrawal periods for meat and offal of 62 for calves and 24 days for small ruminants. Halofuginone is only approved for non-ruminating calves (4–15 days old) unlikely to be slaughtered during or immediately after treatment [124]. In Canada, halofuginone is registered for calves for reducing clinical signs of cryptosporidiosis during the first week of life [125]. The withdrawal periods are 13 days (Europe) and 20 days (Canada) after the last dose of halofuginone for meat and offal from treated calves [125, 126].

A systematic review from over a decade ago [127] on the efficacy of halofuginone for treatment of bovine calves with cryptosporidiosis was inconclusive. However, a more recent systematic review on the same subject [56], with the advantage of further studies to include, considered both prophylactic and therapeutic treatment. The study focused on four outcomes, oocyst shedding, diarrhoea, mortality, and weight gain, and reported beneficial results for two age groups (treatment started over and under 5 days old), although less effective in the older group. The study supported halofuginone compounds as prophylactic treatments, but insufficient studies were available to reach a conclusion regarding therapeutic treatment. The beneficial outcomes from prophylactic treatment were significantly lower incidences of oocyst shedding and a decreased diarrhoea burden in calves receiving treatment, and also an impact on mortality, approaching, but not reaching significance. Weight gain was not found to be affected, but this could be due to the lack of appropriate studies, and some unclear reporting. A scoping review [128] also reported that halofuginone trials consistently resulted in reductions in diarrhoea and oocyst shedding, although also noted that management practices are important.

The systematic review additionally mentioned different problems associated with halofuginone treatment [56]. These include that it should not be administered to calves that have had diarrhoea for more than 24 h or that are already weak or dehydrated, limiting its suitability as a therapeutic treatment, and that as it can be toxic at twice the recommended dose, calves must be weighed in order to determine the correct dose, as overdosing can lead to diarrhoea, visible blood in faeces, decline in milk consumption, dehydration, apathy and prostration [56, 126]. In one case report, increasing the dose of a halofuginone therapy to calves under one month of age by the farmer without veterinary consultation, in order to address anorexia and weight loss, resulted in assumed halofuginone intoxication, with clinical abomasitis and death of some calves [129]. In addition, it has been shown that halofuginone treatment is not effective if there are co-infections with rotavirus or *Salmonella* in calves, speculated to be due to the diarrhoea reducing gut transit time [130].

Paromomycin has also been shown to be effective against cryptosporidiosis in calves, lambs, and goat kids, when used both prophylactically and for treatment [131–133] based on oocyst shedding, clinical disease, and mortality in experimental infections, and supported by results from a field trial in naturally infected dairy calves [134]. Another trial on naturally infected calves indicated that although both halofuginone and paromomycin treatment reduced oocyst shedding similarly (both from day 3) and improved clinical scores, both groups also exhibited symptoms [135]. However, the absence of a control (no treatment) group complicates interpretation of the results.

As with halofuginone, treatment of, or prophylaxis against, cryptosporidiosis in calves with paromomycin raises some problems. One of these is that as paromomycin is an antibiotic there are concerns about its effect on the gut microflora of treated animals, as well as having the potential to induce antimicrobial resistance (AMR) including in the environment as it is poorly absorbed from the gastrointestinal tract and most of the dose is eliminated unchanged in the faeces [121]. However, data on actual concentrations in calf-house manure following treatment are relatively few. In addition, paromomycin should only be given to animals after a cryptosporidiosis diagnosis to limit any negative environmental impacts. Furthermore, although oral absorption of aminoglycosides is low, it may be increased in neonates and when there are enteric infections or pathological changes, suggesting a possibility of nephrotoxicity (kidney damage), especially in dehydrated calves [136].

Treatment for avian cryptosporidiosis, whether respiratory or intestinal, is generally supportive, as treatment trials have given, at best, inconsistent results [137, 138]. Antibiotics may be used as avian cryptosporidiosis may occur together with bacterial infections [139].

### Novel treatments

Given that the pharmaceutical treatments available for livestock cryptosporidiosis are not optimal, the search for a treatment that is more effective and does not come with significant disadvantages remains an active research problem. Among the most advanced novel approaches are targeted molecular therapies. Among these, one of the most studied and most promising is the use of bumped kinase inhibitors (BKI) that target the parasite’s calcium-dependent protein kinase 1, an enzyme that is essential for invading the host cell. Early investigations showed that such an inhibitor reduced oocyst shedding during *C. parvum* infections in calves [140], and later work using three BKIs reported significant improvements in calf cryptosporidiosis as defined by diarrhoea severity, oocyst shedding, and health parameters [141]. More recently, evaluating dosing regimens with a specific BKI were conducted to determine a minimum effective dose. The authors conclude that their results provide strong evidence for advancing towards a preclinical candidate for treatment [142]. A further family of enzymes that has been identified as potential target for treatment of cryptosporidiosis is phosphatidylinositol-4 kinase (PI4K), and reduced oocyst shedding and reduced diarrhoea has been shown in calves [143]. In addition, promising results have been obtained with lysyl-tRNA synthetase inhibitors, which reduced oocyst shedding to below detection levels and also reduced diarrhoea in calves [144].

While these drugs remain in pipeline stages, and often with an intended endpoint of human infection, there has also been considerable research directed towards probiotics, prebiotics, plant-derived compounds, and nutritional supplements, either to treat cryptosporidiosis or provide supportive therapies.

Many of the studies directed towards treatment are in vitro, which, while providing useful initial data often over-predict the usefulness of a compound, with promising in vitro results not being replicated in in vivo trials. There are several reasons for this lack of translation, including the host-parasite relationship being difficult to reproduce in in vitro culture systems and that the disease ecology is more complex than can be replicated in an experimental in vitro system. In many studies, particularly on potential phytochemical compounds, the experiments expose parasites to concentrations that cannot be achieved orally in calves, that are likely to be metabolized or passaged too rapidly to have the required contact time. This is especially relevant for plant compounds being degraded before the small intestine, potentially due to microbial metabolism. In addition, studies often suggest a requirement for concentrations that would be economically and practically difficult to achieve in livestock. Further information can be obtained from individual experimental reports, too numerous to be detailed here, or from reviews (e.g [145, 146]),.

For supportive, rather than curative, therapy using prebiotics or probiotics, evidence tends to be weak, although modest improvements in the severity of diarrhoea or illness severity have been reported, possibly due to stabilization of gut microbiota, modulation of inflammation, or improved epithelial repair and mucosal immunity. Nevertheless, the results tend to be mixed, with some investigations finding that probiotic treatment may actually negatively impact weight gain [147].

It is clear that there are important gaps in the treatment of livestock cryptosporidiosis, with advantages and disadvantages for both paromomycin- and halofuginone-based treatment or prophylaxis, including that their use is geographically restricted. Although new possibilities are being actively investigated, and some approaches seem promising, it is clear that in vitro results often fail to deliver in vivo*.*

## Future challenges and important gaps in knowledge and implementation of tools for cryptosporidiosis control in livestock

Although the development and production of a vaccine against bovine calf cryptosporidiosis, along with the availability of on-site (pen-side) diagnostic tests, have been important steps forward in control of cryptosporidiosis, it is pertinent to consider future challenges that may lie ahead.

### Changes in farm management

In general, there is a move towards larger, more intensive farming in many parts of the world. Agricultural intensification is considered to be a key move in modernization, with higher densities of animals, more reliance on feed rather than grazing, and larger yields per hectare. In Europe, for example, there are now fewer dairy farms and almost 80% of milk is produced in intensive farming [148]. Although on a global scale, the small-holder farms predominate, representing around 85% of all farms [149], future trends suggest that declining rural populations will drive widespread consolidation towards larger farms, including in Asia and Africa [150]. In general, larger farms have long been associated with higher *Cryptosporidium* infection rates in livestock (e.g [151, 152]). Factors that are potentially associated include: high-stocking density enabling more rapid spread between animals and unit [93, 153], housed feeding systems creating environments for persistence and spread of the oocysts, and a greater faecal load in the environment promoting contamination. Thus, should the trend continue of fewer, larger farms, we may expect that cryptosporidiosis could also become a greater problem among livestock, potentially with overflow to people.

### Climate change

Weather conditions are known to affect the spread of cryptosporidiosis, and some aspects of climate change are likely to increase transmission in particular regions [154]. While hotter, drier conditions are likely to reduce the survival of oocysts in the environment, such conditions may also mean that the oocysts, and other pathogens, become concentrated in smaller volumes of water [155], potentially increasing infection risk for animals. Although freeze-thaw cycles are known to inactivate *Cryptosporidium* oocysts in soil or on pasture [156, 157], warmer winters may result in grazed pastures remaining contaminated with viable oocysts until spring, when young animals may be released on to them. Heavy precipitation associated with climate change can wash livestock faeces from pastures, outdoor pens, and manure heaps into streams and ponds, and extreme rainfall may even overwhelm farm drainage systems, spreading water containing oocysts into drinking water supplies or grazing areas. In cool, damp conditions, oocysts are known to survive better [9], and wet bedding and muddy conditions are likely to assist in maintaining viable oocysts in areas where livestock are kept, whether inside or out. In extremely wet conditions, livestock may be held inside for longer periods, forcing animals into crowded house, which will also increase transmission risk.

### Expanding distribution of different species and genotypes

Our knowledge on the distribution of different species and genotypes of *Cryptosporidium* has increased vastly over the past decade and continues to expand as more data are collected. One question that has been posed is the apparent paucity of infections with zoonotic *C. parvum* subtypes among both livestock and people in many countries in sub-Saharan Africa [13]; some extensive studies have reported not finding any *C. parvum* in livestock at all, and in people only *C. hominis* or those subtypes of *C. parvum* that are not considered zoonotic. It has been speculated that this may reflect extensive, rather than intensive, farming, and lack of susceptibility of local cattle breeds maybe potential reasons. However, it has also been speculated that zoonotic *C. parvum* evolved in cooler, damper climates, and a combination of globalisation and climate change may provide an entry point for expansion. Further knowledge on the different species and subtypes, and their association with different properties such as host-specificity and virulence will continue to be important in control of cryptosporidiosis in both people and livestock.

## **C**onclusions and summary of knowledge gaps

Although there have undoubtedly been advances in our knowledge of *Cryptosporidium* and cryptosporidiosis in livestock over the last five decades, there remain clear gaps in our knowledge and in our tools to control this disease (Table 1). As cryptosporidiosis all too frequently spills over from animals to people, particularly regarding *C. parvum*, this is a public health issue as well as one of veterinary health. We hope that this article, and its accompanying chapter in the DISCONTOOLS database [5] will serve as an inspiration to researchers, both in academia and in industry, to focus their attentions to where there are obvious gaps in our knowledge and where tools to control this disease remain inadequate.

**Table 1:** Currently available diagnostic methods, pharmaceutical interventions, vaccines, management strategies; advantages, limitations, implementation status, and knowledge gaps

	Interventions	Advantages	Limitations	Implementation status	Knowledge gaps
Diagnostic methods	Microscopy-based tools for detection of oocysts in faeces	Requires only low cost, relatively basic equipment	Low sensitivity; requires experienced operator; time consuming. Cannot differentiate between species.	Widely used by diagnostic laboratories.	Will digital microscopy and computer-based screening methods to identify oocysts/cysts in faecal samples enhance diagnosis?
	Immuno-chromatographic lateral flow (ICLF) tests	Portable; rapid and differential results; can be used on farm.	More expensive than microscopy; often not be quantitative. Short shelf-life. May not be differential or detect all species.	Used on farms and by veterinarians sometimes by farmers. Available in Europe but not yet in the USA.	Unknown which species can be detected by each test.
	DNA-based diagnostics and typing tools	Very sensitive, can identify species. Typing tools can help to track infection sources.	Expensive and requires specific equipment; slower time to result than ICLF tests.	Used by reference laboratories and for research.	Genome-targeted enrichment technologies need further development; whole genome sequences need to be generated for more *Cryptosporidium* species.
Vaccines	Bovilis Cryptium	Reduces severity of, and deaths due to, cryptosporidiosis.	Relies on good colostrum management and feeding of follow-on milk. Does not prevent infection or environmental contamination	First licensed in Europe in 2023 and starting to become elsewhere in the world.	Requires further independent scientific scrutiny to assess efficacy in working farms. Evaluations of vaccine efficacy in other hosts and for other *Cryptosporidium* species required.
Pharmaceutical interventions	Halofuginone lactate	Halofuginone can be used prophylactically.	Cannot be given to dehydrated animals due to risk of toxicity.	First drug licensed for use against cryptosporidiosis (2004 in Europe). Widely used internationally, although not in the USA.	Evidence is needed to show if halofuginone, which is only licensed for use in cattle, will also work in sheep, goats or other animals.
	Paromomycin sulphate	Paromomycin can be used as a treatment.	Requires confirmation of infection before starting treatment. Poorly absorbed and thus environmental contamination with the antibiotic results from treatment	Came onto the market in Europe in 2018 and is becoming available in other parts of the world.	Although its mode of action is well characterised for bacteria, how paromomycin enters *Cryptosporidium* and its mode of action requires further elucidation.
Management strategies	Farm management and hygiene	Effective farm management and hygiene practices reduce the impact of cryptosporidiosis.	Farmers are limited by their infrastructure.	Practices vary widely.	Lack of well powered studies to evaluate the effect of different farm management and hygiene practices.
	Colostrum management	Provides passive immunity to naive neonatal animals until they develop their own immune responses.	Not all dams produce good-quality colostrum; pooled colostrum may be a risk for spread of other pathogens. Neonatal ruminants require sufficient good quality colostrum directly after birth.	It is widely accepted that good colostrum management is essential but implementation on individual farms can vary.	There is lack of knowledge of protective components within colostrum and how colostrum aids in development of natural immunity.
	Disinfection	Effective disinfectants reduce environmental contamination with viable oocysts.	Many on-farm disinfectants have only poor efficacy at inactivating *Cryptosporidium* oocysts.	Disinfectants widely used on farms, but not all are effective against *Cryptosporidium*.	Good comparable data for different chemical compounds or products is lacking.
	Slurry and manure management	Effective slurry and manure management will inactivate oocysts and reduce environmental contamination with viable oocysts.	The effectiveness of slurry and manure management is impacted by temperature; longer storage times may be needed to achieve oocyst inactivation	Slurry and manure management practices widely used, but vary over time and between farms.	Better evidence-based scientific data needed to understand oocyst inactivation dynamics within a midden.

In particular, we would like to highlight our knowledge of the basic biology of some species of *Cryptosporidium* in livestock remains diffuse. We know that different *Cryptosporidium* species in the same host animals may behave differently with regard to pathology, oocyst shedding, and clinical disease, but without a clear understanding of which biological mechanisms are associated with these differences. At the same time, the same *Cryptosporidium* species in different, but related, host species may cause different diseases. Similarly, the reasons for geographical differences in distribution – such as the apparent scarcity of zoonotic *C. parvum* in both livestock and people in many sub-Saharan African countries remains difficult to understand, although different reasons have been proposed.

Diagnostic tools are probably one area where the most successful developments have been made with *Cryptosporidium* oocyst-specific antibodies labelled with fluorogens enabling IFM for detection. Similarly, molecular tools are now available that enable sensitive and specific diagnostics, along with enabling the epidemiology and transmission routes to be dissected, along with tracking of outbreaks at the sub-species level. Although widely adopted, validation at the international level is lacking, and species-specific pen-side tests would be a valuable addition. Improved bioinformatics pipelines to process WGS data have the potential to improve our understanding of virulence drivers and other aspects of interest for control.

The first vaccines against bovine cryptosporidiosis are still new in our armoury for control of *Cryptosporidium* in livestock and current results are promising, but there are clear limitations and room for improvement. Although clinical improvements are of value, as full prevention of oocyst shedding is lacking, the transmission risk remains, including to people. Additionally, the lack of evaluation criteria remains a challenge and critical endpoints must be agreed upon for integrated control programmes to be evaluated. Other challenges are regional availability and that the vaccine is limited solely to cattle; extending the same vaccine approach to small ruminants could be of value, but a new strategy will be needed for a vaccine for poultry or avian cryptosporidiosis.

Even with a vaccine, on-farm measures against cryptosporidiosis in livestock remain important. Although the importance of colostrum is widely acknowledged, detailed data on, for example, the efficacy of pooled colostrum, colostrum replacers, or colostrum supplementation is missing. Standardised protocols are necessary to compare the efficacy of disinfectants at inactivating oocysts in the environment, but are largely lacking, and the inactivation dynamics of oocysts in slurry and manure are not properly understood. Experiments providing reliable data would be of value. Finally, understanding the motivation of farmers to implement or not biosecurity measures to protect both livestock and farm workers or visitors from cryptosporidiosis are not properly understood in different settings.

Although two pharmaceuticals for early treatment during clinical cryptosporidiosis or for prophylactic use are approved in some regions and for some livestock species, they are not approved globally nor for all livestock that may be infected. While both seem to have some important effect on clinical cryptosporidiosis in ruminants, including decreasing diarrhoea and oocyst shedding, both also have some disadvantages. These include, but are not limited to, the potential for toxicity with overdosing (halofuginone) and the potential for effects on gut microflora and potential for inducing AMR in the environment due to high a high proportion being eliminated in the faeces (paromomycin). Neither of them has been approved for use against cryptosporidiosis in pigs or poultry. There is thus a clear need for further development of pharmaceuticals for which registration authorities are convinced that the benefits outweigh any disadvantages such that geographical limitations are addressed, and that also may tackle cryptosporidiosis in all livestock species. Although research in this area is clearly active, progressing to meaningful in vivo studies should be emphasized.

With so many different gaps to fill for optimal tackling of livestock cryptosporidiosis, we welcome the opportunity to encourage research to improve our basic knowledge on this parasite and to address the current deficiencies in our tools for control of this parasite.

## Data Availability

This article is based on a DISCONTOOLS research gaps exercise on *Cryptosporidium* infection in livestock. All authors participated in providing information for the database.
